# Experimental study of gamma-ray attenuation capability of B_2_O_3_-ZnO-Na_2_O-Fe_2_O_3_ glass system

**DOI:** 10.1038/s41598-024-68941-3

**Published:** 2024-08-19

**Authors:** Mohamed Elsafi, M. I. Sayyed, Taha A. Hanafy, Chaitali V. More, Ali Hedaya

**Affiliations:** 1 Physics Department, Faculty of Science, Alexandria University, Alexandria, 21511 Egypt; 2 Department of Physics, Faculty of Science, Isra University, Amman, Jordan; 3 Department of Physics, Faculty of Science, University of Tabuk, Tabuk, Saudi Arabia; 4 Department of Physics, Dr. Babasaheb Ambedkar Marathwada University, Chhatrapati Sambhajinagar, 431004 India; 5 Department of Environmental Studies and Research, Institute of Graduate Studies and Research, Alexandria University, Alexandria, Egypt; 6 Department of Physics and Technical Sciences, Western Caspian University, Baku, Azerbaijan

**Keywords:** Lead oxide, Archimedes’ principle, Radiation shielding glasses, Attenuation factors, Physics, Nuclear physics

## Abstract

In the present work, a glass system with developed composition consisting of B_2_O_3_, ZnO, Na_2_O and Fe_2_O_3_ samples has been investigated. Glass samples were prepared using the melt quenching method and the density of the system was measured using Archimedes’ principle. Spectroscopic analysis using a gamma source and a high-purity germanium detector at four energies of 0.0595, 0.6617, 1.173, and 1.333 MeV emitted from Am-241, Cs-137, and Co-60 were used to determine the attenuation parameters of present glass composites. The sample containing 45 B_2_O_3_ + 10 Na_2_O + 40 ZnO + 5 Fe_2_O_3_ (coded BNZF-4) had the highest mass attenuation coefficient (MAC) value at all the energies discussed compared to the other composites. Whoever, the BNZF-1 sample had the lowest value at all ranges of energies. The transmission factors (TF, %) of the manufactured samples were calculated, at 0.0595 MeV (TF, %) values are 32.6429 and 6.4612 for samples BNZF-1 and BNZF-4, respectively. The statistical results demonstrated significantly better to increase the ZnO concentration in the sample, where the percentage of zinc oxide inside the prepared glass samples has the following direction BNZF -4 > BNZF -3 > BNZF -2 > BNZF -1. The significance of this study is that transparent, environmentally harmless glass composites with relatively high density have been prepared that can be used as shielding materials against gamma rays, especially at low energies.

## Introduction

Owing to advancements in technology, people are exposed to the risks of ionizing radiation through normal background radioactivity, mining, milling, nuclear power plants that use synthetic radioactive isotopes, space exploration, nuclear research, and other areas. It is crucial to note that radiation exposure can cause a variety of health problems, among the most significant of which include carcinomas, genetic disorders, and tissue damage. It's also crucial to remember that ionizing radiation can alter the physical properties and chemical structure of soil, rock, and water, which can have a negative effect on biodiversity and the ecosystem. This requires careful investigation and effective protection and shielding techniques because they pose significant risks to humans^1–3^.The protection can be attained by using efficient shields, which necessitates researching all aspects of possible shielding substances, including their mechanical attributes, various radiation attenuation features, anticipated energies, expenses and accessibility of substances, no difficulty in manufacturing, performance, and the probability of affordable prices^4–6^.

A crucial characteristic is the degree of resistance of the shielding material against potential harm induced by being exposed to ionizing electromagnetic radiation, together with ensuring that the shields are non-toxic. Along with a large rays-absorption cross-section, the efficient shielding substance must also induce a considerably greater strength attenuation of the incoming rays throughout a narrow penetration depth (thickness)^7^. Various kinds of substances are frequently employed for this purpose, in accordance with the intended use. For example, because concrete is useful and excellent at attenuating X-rays, it is widely used as the absorber along the exterior walls of X-ray rooms. Concrete may serve as a perfect material in certain situations, but alternative materials are occasionally required because it can crack easily and lose moisture when exposed to radiation for an extended period^8,9^. Glasses by adding metallic oxides to their formulation, are capable of functioning as radiation shielding materials. Since their high density raises the density of the glass materials, which usually corresponds to higher shielding properties, heavy metal oxides are usually among the most efficient^10–14^. Moreover, well-known techniques like melt quenching are applicable to create glasses. Because glasses are inexpensive to manufacture, scientists are more inclined to employ them as alternative components for materials that need radiation shielding^15^.

When manufacturing our glass materials, a few factors will be taken into consideration. These include the need for a large mass density as well as excellent transparency to the visible portion of the electromagnetic range to be able to provide beneficial shielding properties and guarantee a notably significant interaction probability between the glass and photons. Consequently, this elevated contact probability means that the energy of ionizing rays will be much reduced and the rays' capacity to pass through glass will be eliminated^15,16^. To improve radiation shielding, one of the strategies is to increase the glass density. It was reported that, glasses based on borate have low viscosity, great mechanical strength, short glass transition temperature, high chemical durability, and clear transparency. They are also cost-effective materials. Owing to these characteristics, glasses based on borate have gained attention for a wide range of uses, such as biomedical, shielding, industrial, and several other uses^17–19^. By adding metal oxides as network modifiers such as ZnO, Na_2_O, Fe_2_O_3_, borate glass can be tested for its radiation shielding properties^20^. Borate-based glasses are gaining a lot of attention as radiation shielding materials and considered a hot topic in the discipline of radiation protection safety. Glasses made of borate could have superior shielding properties as their density could be adjusted. Using heavy-duty rare earth oxides and heavy earth oxide metal oxides in glass samples are a simple way to increase glass density^21–23^.

In this work, our goal was to manufacture non-toxic and cost-effective glass samples of various compositions using a melt quenching procedure (85-x) B_2_O_3_ + 10 Na_2_O + (x)ZnO + 5 Fe_2_O_3_, where x = 10, 20, 30 and 40 wt %. It is also an attempt to mitigate the harmful effects of radiation on humans and the environment and ensure the benefit of ionizing radiation in the long term without exposure to serious harm.

## Materials and methods

### Glass manufacturing

To prepare the glass samples under investigation, some oxides were used, such as ZnO, Na_2_O, Fe_2_O_3_, and B_2_O_3_. Four samples of zinc sodium borate glasses were manufactured with different ratios among them as shown in Table.1 and the ratio were variable between B_2_O_3_ and ZnO and constant for Na_2_O, Fe_2_O_3_. The samples were Manufactured according to the annealing technique, the material has been mixed perfectly well and conveniently and placed in a crucible of aluminum and then entered an electric oven at a constant temperature of 950 Celsius for a full 60 min. The other stage is to pour the mixture of molten glass into stainless-steel mold and put it in a separate electric oven at a temperature of 300 for almost 180 min to eliminate internal stresses.

**Table 1:** Chemical composition of the fabricated samples and their density (g/cm^3^).

Glass name	Composition (mol %)				Density (g/cm^3^)
	B_2_O_3_	Na_2_O	ZnO	Fe_2_O_3_	
BNZF -1	75	10	10	5.0	2.7677
BNZF -2	65	10	20	5.0	2.9632
BNZF -3	55	10	30	5.0	3.1809
BNZF -4	45	10	40	5.0	3.4251

The density of each sample is an important parameter in our work. So, to precisely determine the density of the six samples, we used a very simple and correct method of calculating the density of each sample. The following equation, which uses Archimedes’ is used to calculate the density of the manufactured glasses, uses the (W_a_) and (W_L_) values as symbols for the weight of the glasses in liquid and air. Correspondingly, when utilizing water as an immersing liquid, the ρ_L_ value represents the density of the immersed liquid, which is taken as 1 g/cm^3^^24,25^.

1 $$\rho =\frac{{W}_{a}}{{W}_{a}\text{ - }{W}_{L}}{\rho }_{L}$$

## Experimental procedures

**Figure 1:**
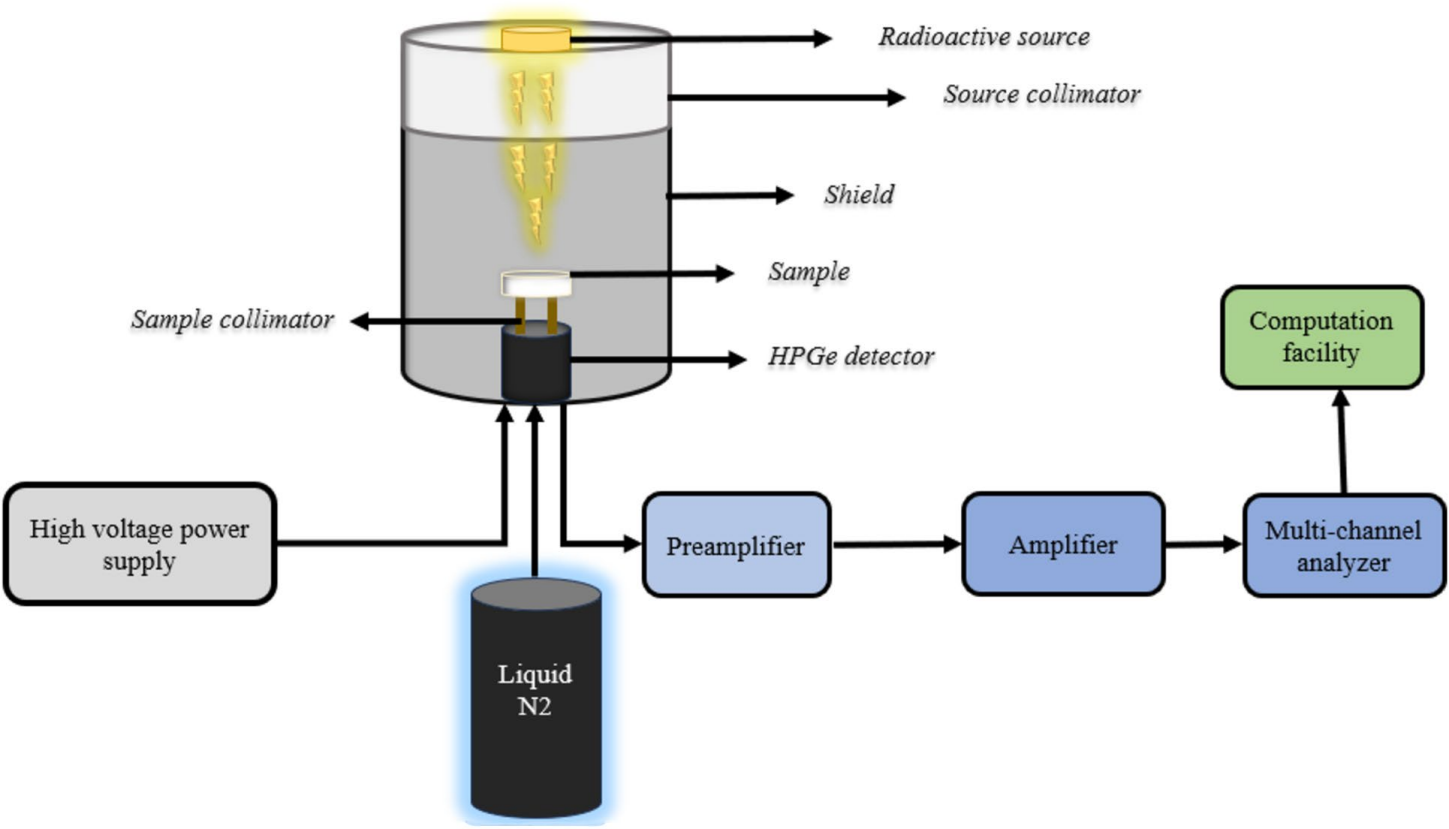
The HPGe diagram setup.

The ionizing γ-ray sources Am-241, Co-60, and Cs-137 were identified using an HPGe (high-purity germanium semiconductor detector) with a 24% relative efficiency in this research. The energy range covered by these sources is 60 to 1333 keV. The glass sample was inserted at an appropriate location between the gamma source and the HPGe, as shown in Fig. 1. It displays the experimental setup diagram of attenuation factor calculations., With a lead collimator positioned between the gamma source and the HPGe-detector, the measurements were carried out using the narrow beam technique. The count rate was measured in the case of the sample (A) and in its absence (A_0_), and those results were recorded Enabling us to identify linear attenuation coefficient (cm^−1^) as well as some important parameters in our work^26–28^

2 $${(\mu )}_{(c{m}^{-1})}=\frac{1}{x}ln\frac{{A}_{0}}{A}$$

where x is the glasses sample thickness, depending on I and Io calculations, the other essential shielding-related parameters, such as the radiation protection efficiency (RPE %), half-value thickness (Δ_0.5_, cm), and lead's equivalent thickness (Δ_eq_, cm), can be expressed using the following formulae^29–31^.

3 $$RPE \, \% \, = \left[ {1 - \frac{A}{{A_{0} }}} \right] \times 100$$

4 $${(\Delta \_0.5)}_{(cm)}=\frac{ln(2)}{\mu }$$

5 $$\left( {\Delta_{eq} } \right)_{{\left( {cm} \right)}} = \frac{{X_{{\left( {cm} \right)}} \ln \left( {\frac{{A_{0} }}{A}} \right)_{lead} }}{{ \ln \left( {\frac{{A_{0} }}{A}} \right)_{glass} }}$$

## Result and Discussion:

Table 1 lists the names, densities, and chemical constituents for each chosen glass system. Table 2 shows the results of linear attenuation coefficients, LAC, at certain gamma energies viz., 0.0595, 0.06617, 1.173 and 1.333 MeV acquired with the experimental and theoretical methods. Experiments were performed with the HPGe detector. The renowned XCOM programme was used to confirm the results that were so acquired. The LAC data were also further utilized to compute additional shielding parameters namely mass attenuation coefficient (MAC), half value layer (HVL), mean free path (MFP), tenth value layer (TVL) and radiation protection efficiency (RPE). Additionally, an assessment of the manufactured glass materials’ radiation shielding effectiveness (RSE) and transmission factor (TF %), have been determined.

**Table 2:** Linear attenuation coefficients (LAC, cm^−1^) for studied glasses.

LAC, cm^−1^												
Energy (MeV)	BNZF-1			BNZF-2			BNZF-3			BNZF-4		
	XCOM	Exp	Dev%	XCOM	Exp	Dev%	XCOM	Exp	Dev%	XCOM	Exp	Dev%
0.060	1.1195	1.0923	2.49	1.6013	1.5832	1.14	2.1379	2.0383	4.89	2.7394	2.6052	5.15
0.662	0.2080	0.2001	3.94	0.2222	0.2381	−6.67	0.2381	0.2253	5.69	0.2559	0.2467	3.72
1.173	0.1582	0.1511	4.72	0.1688	0.1591	6.09	0.1805	0.1728	4.48	0.1938	0.1889	2.57
1.333	0.1482	0.1492	−0.68	0.1581	0.1511	4.62	0.1691	0.1614	4.79	0.1815	0.1896	−4.28

**Figure 2:**
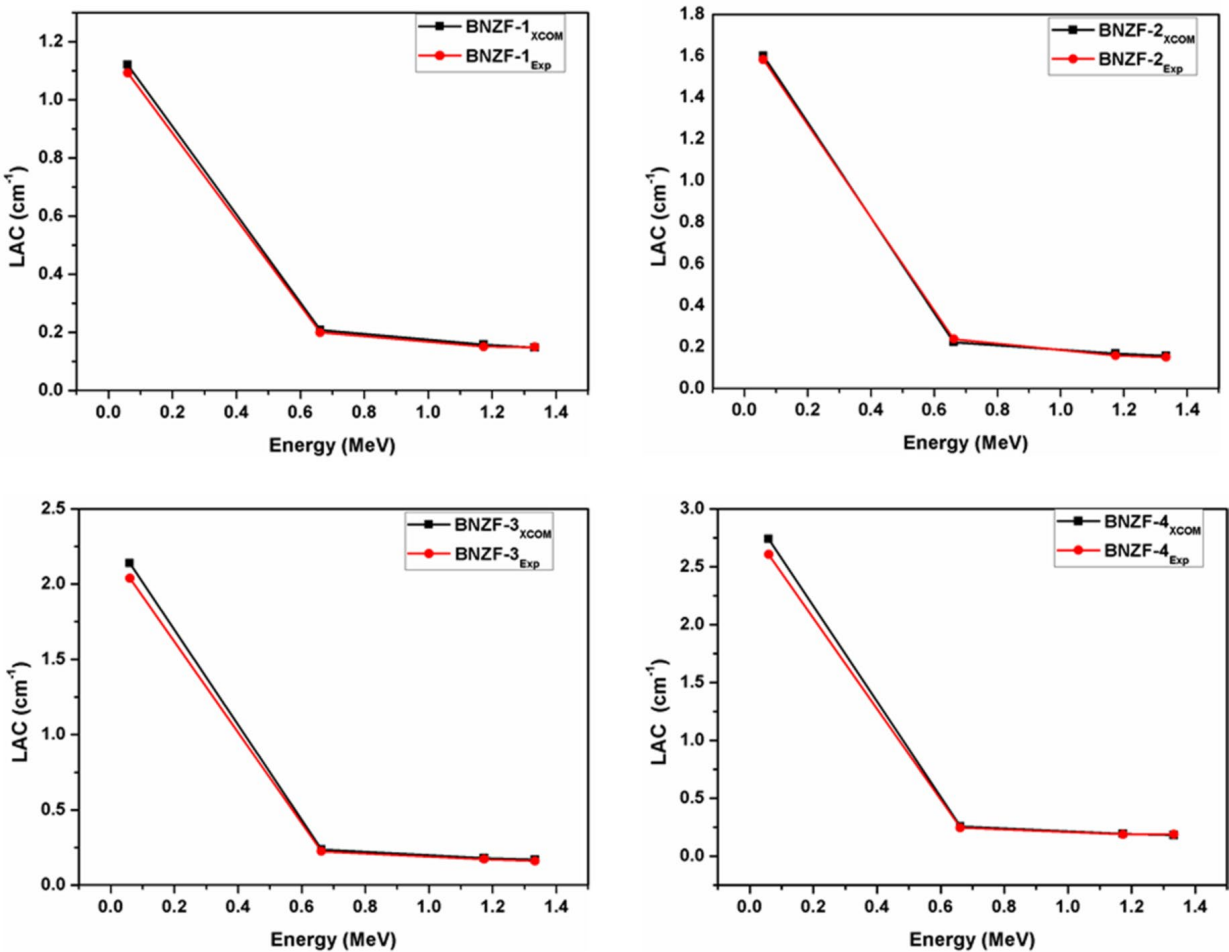
Variation of LAC (cm^−1^) for studied glasses for 0.0595–1.333 MeV.

Table 2 presents the relative deviations of LAC values for glass samples obtained from XCOM programme and experiments. It is seen that the relative differences between the XCOM programme and the LAC values obtained from trials are negligible. For example, the theoretical value of 1.1195 validates the experimental value of 1.0923 for BNZF-1 glass at low energy of 0.05595 MeV. Additionally, the XCOM value of 0.1482 confirms the experimental value of 0.1492 for the same glass system at higher energy (1.333 MeV). For the glass samples under investigation, the range of experimental and theoretical deviations is from −6.67 to 6.09. Figure 2 illustrates how the LAC values of the chosen glass samples varied across the photon energy range of 0.0595 MeV to 1.333 MeV. It is clear from this that LAC is dependent upon the chemical composition of the samples as well as the incoming photon energy. The sample BNZF-4 exhibits higher values of LAC than the others because it has the largest density and greater weight fraction of B_2_O_3_ (Table 1). After a steep decline from 0.0595 to 0.6617 MeV, there is a little variation in LAC values. The likelihood of interaction is determined by the atomic number (Z^n^), where the exponent n fluctuates from 4 to 5, and the dominance of the photoelectric effect at lower energy, in which the interaction cross section depends on energy as σ_Ph_ ~ E^−7/2^. At intermediate energies, the Compton effect (σ_Com_ ~ E^−1^) predominates. The attenuation levels at these energies were the same in all samples, as indicated by the LAC values. The reason for this is that Compton scattering has a linear dependence on atomic number, Z^32,33^.

**Figure 3:**
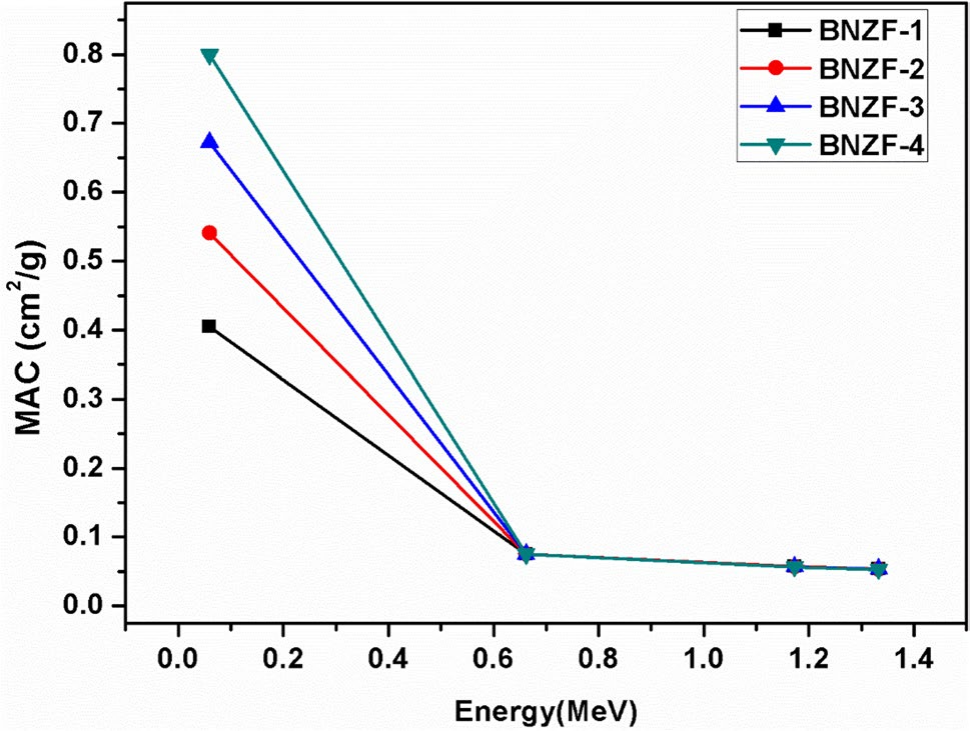
Variation of MAC (cm^2^/g) for studied glasses for 0.0595–1.333 MeV.

The mass attenuation coefficient (MAC) quantifies the average number of interactions between light photons and matter in a certain mass per unit area thickness of the substance under investigation^33^. Figure 3 displays the glasses’ MAC values; the photon energy extends from 0.0595 to 1.333 MeV. It was found that the components of the glasses have a significant impact on MAC values; MAC values behave in line with B_2_O_3_ levels. Other constituents Na_2_O, ZnO and Fe_2_O_3_ have same proportions for all the glasses. The MAC is in the following order: BNZF-4 > BNZF-3 > BNZF-2 > BNZF-1. The energy of the incident photons shows a consistent pattern across all glasses in the MACs. For all glasses, MACs show the same trend in the energy of the incident photons, and it has similar behaviour as that of LAC.

**Figure 4:**
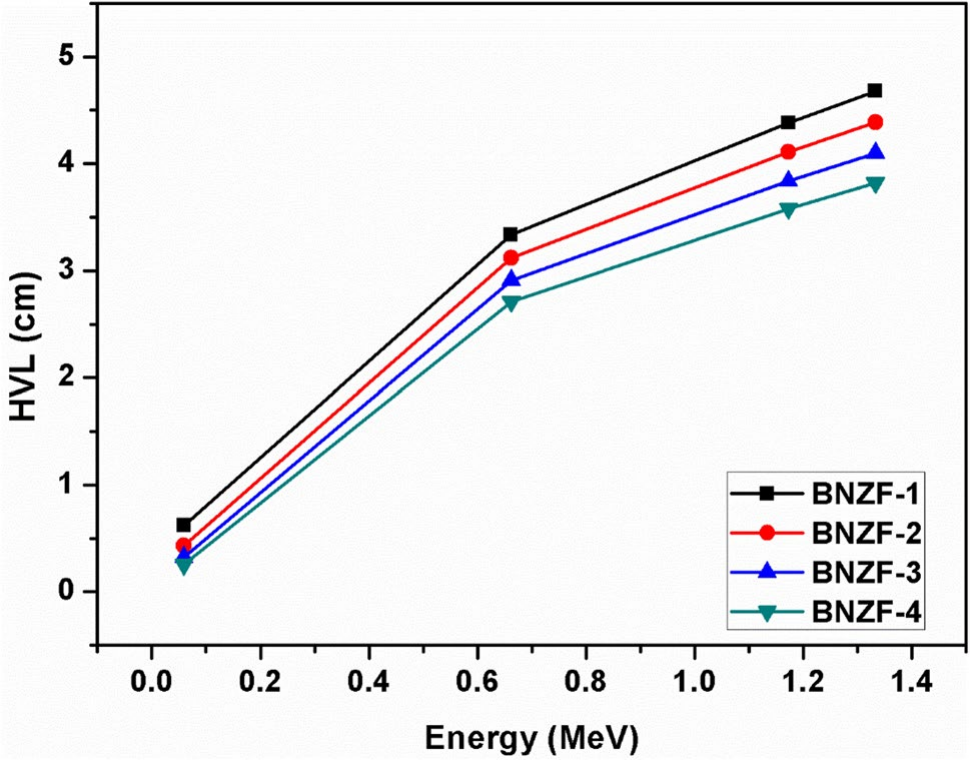
Variation of HVL (cm) for studied glasses for 0.0595–1.333 MeV.

**Figure 5:**
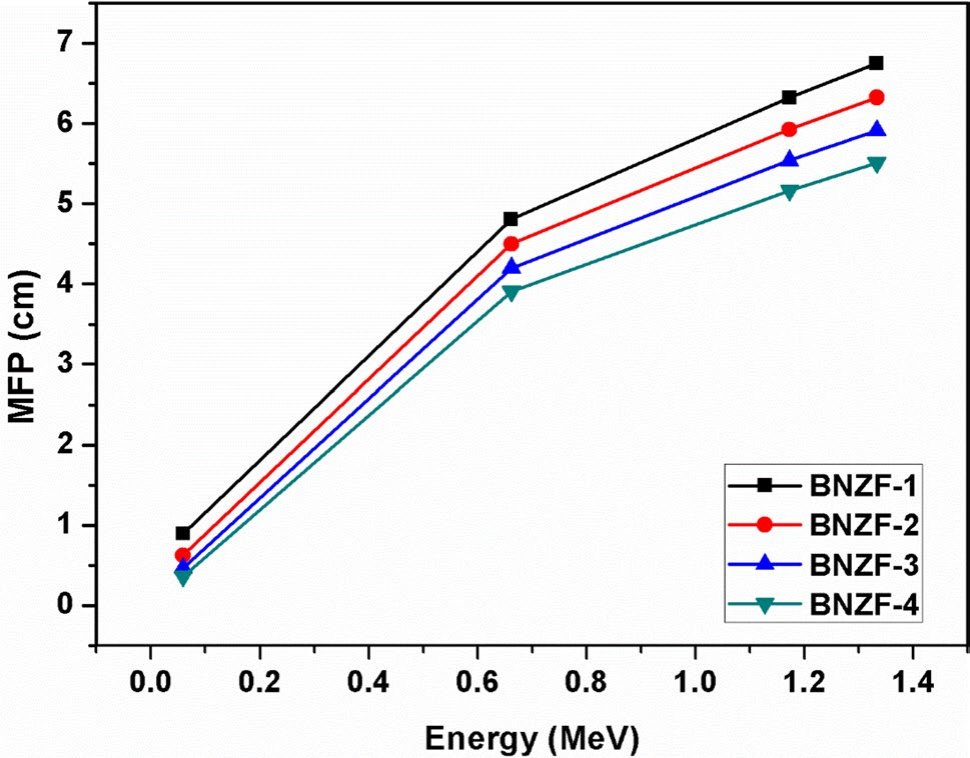
Variation of MFP (cm) for studied glasses for 0.0595–1.333 MeV.

**Figure 6:**
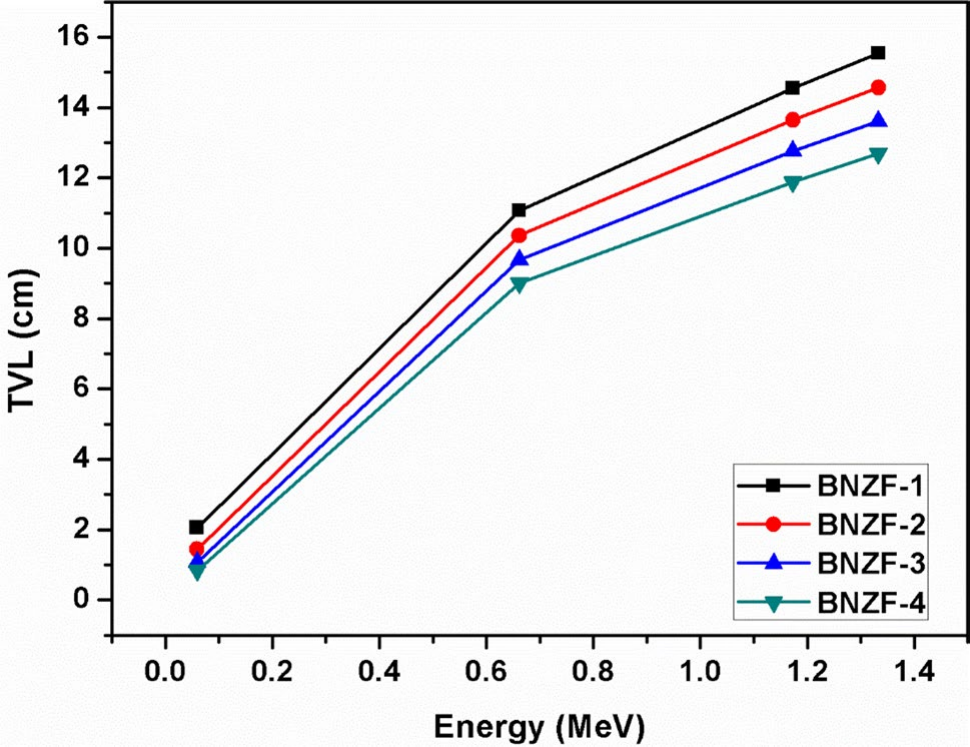
Variation of TVL (cm) for studied glasses for 0.0595–1.333 MeV.

The half value layer (HVL), tenth value layer (TVL), and mean free path (MFP) variations as a function of the source photon energy are displayed in Figs. 4,5 and 6. These are important characteristics that provide the necessary material thicknesses at certain energy and the material's ability to shield. These variables exhibit the exact opposite pattern of variation from LAC, that is, a rising tendency with incident energy. The lowest values of these parameters across all samples are found at 0.0595 MeV. At lower energy, these thicknesses fall into the following ranges: 0.253–4.678 cm (HVL); 0.365–6.748 cm (MFP); and 0.841–15.539 cm (TVL). The values of these characteristics were found to increase with the following trend BNZF-4 < BNZF-3 < BNZF-2 < BNZF-1 among the selected samples. This indicates that BNZF-4 is a superior radiation shield among the glasses under investigation. This is explained by the fact that BNZF-4 has the largest density of all the materials under study, which reduces values for HVL, TVL, and MFP and raises the likelihood of interaction. These outcomes are consistent with the earlier research^34–36^.

**Figure 7:**
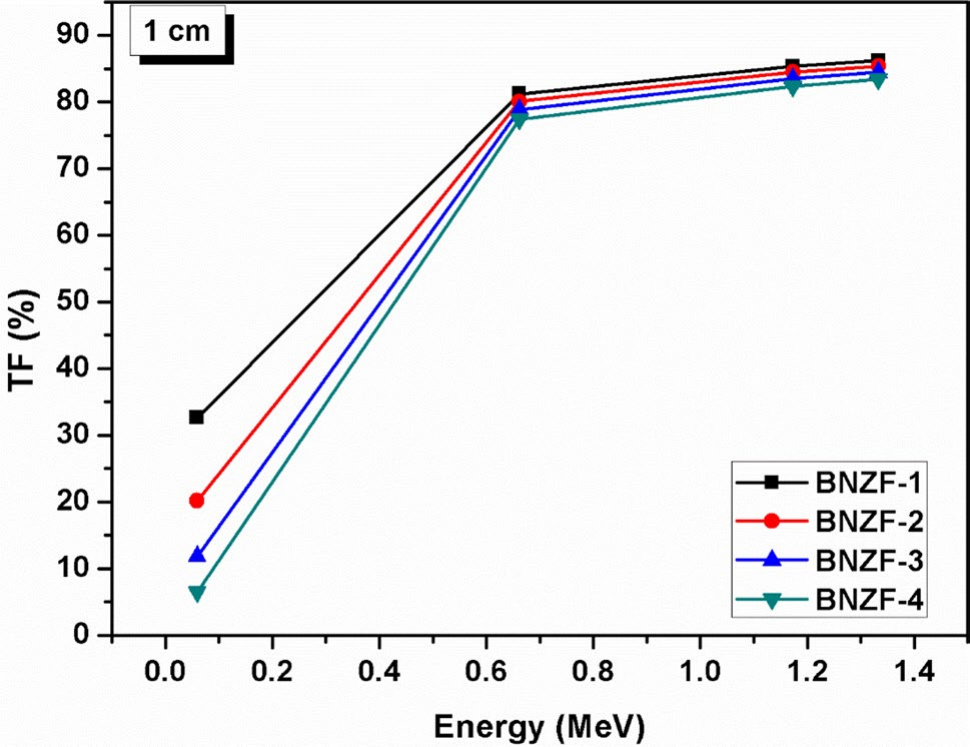
Variation of TF (%) of studied glasses for 0.0595–1.333 MeV at 1 cm.

**Figure 8:**
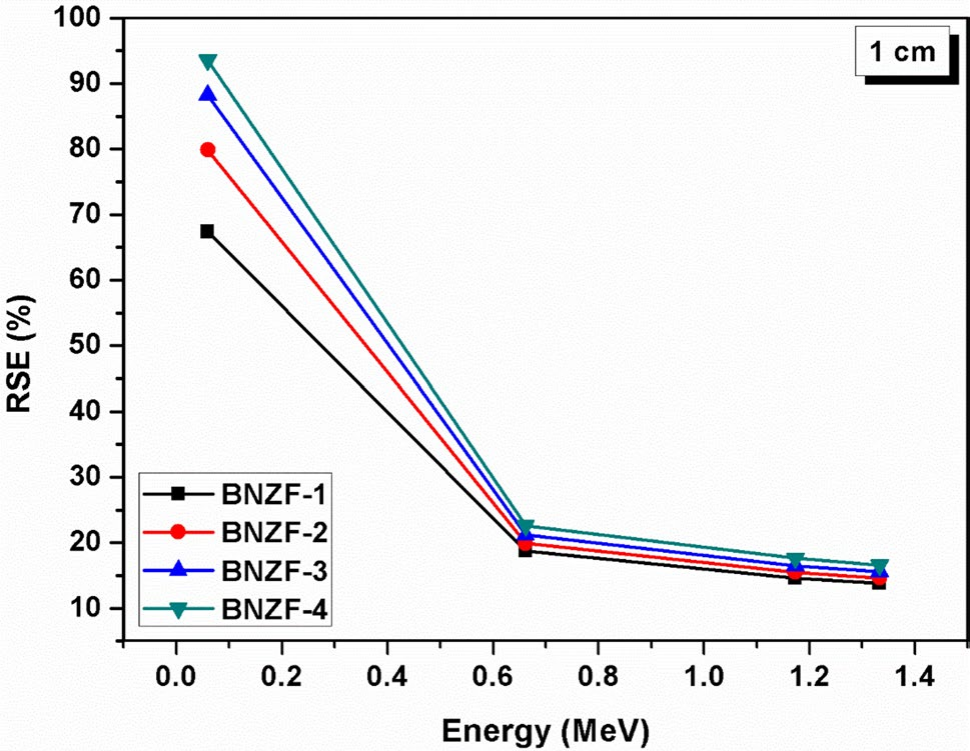
Variation of RSE (%) of studied glasses for 0.0595–1.333 MeV at 1 cm.

Figure 7 depicts variation of transmission factor (TF, %) versus energy for 1 cm thickness for the glasses under investigation. The trend of variations in TF levels is comparable to that of energy. TFs have lower values at lower energies and increase with increased photon energy. Sample BNZF-1 has TF of 32.6429 whereas BNZF-4 has TF value 6.4612 at 0.0595 MeV. At higher energy of 1.333 MeV, BNZF-1 and BNZF-4 glasses have TFs 86.2273 and 83.4024 sequentially. The effectiveness of a shielding material is determined by several parameters, one of the crucial parameters is its radiation protection efficiency, RSE (%) of the investigated glasses as a function of energy have been portrayed in Fig. 8 at 1 cm. An inverse relation is clearly observed between energy and RSE^33^. This declining tendency is brought on by higher energy photons’ greater penetrating power, which lessens ability of these glasses to absorb/block incoming radiation. At 0.0595 MeV, BNZF-1 glass has RSE 67.357% and other glasses have RSE in order of 79–93%. This indicates that the glasses under study are very good at attenuating the lower-energy photons. Among the selected glasses, BNZF-4 glass has shown greatest radiation shielding efficiency.

## Conclusion

In the presented investigation a high-purity germanium detector was used for assessing the linear attenuation coefficient of four novel B_2_O_3_, ZnO, Na_2_O, and Fe_2_O_3_ glass components coded (BNZF-1 to BNZF-4) at four various energy ranges: Am-241 (0.0595 MeV), tansmetCs-137 (0.6617 MeV), and Co-60 (1.173 and 1.333 MeV). The results indicated that the linear attenuation coefficient values of the glass samples decreased by the following trend BNZF -4 > BNZF -3 > BNZF -2 > BNZF -1. Moreover, the partial density jumped when ZnO concentration grew in relation to B_2_O_3_. The manufactured glasses’ density is increased between 2.7677 and 3.4251 g/cm^3^ when ZnO is substituted for B_2_O_3_, with an increase of 10–40 mol% for ZnO. Likewise, the manufactured BNZF glasses’ ability to attenuate gamma radiation is enhanced when ZnO is substituted for B_2_O_3_. It is obvious that the glass components have a significant impact on the mass attenuation coefficient MAC values; MAC values behave in line with ZnO levels as the following order: BNZF-4 > BNZF-3 > BNZF-2 > BNZF-1, ranging from highest values 0.802 cm^2^.g^−1^ to lowest value 0.406 cm^2^.g^−1^ at 0.0595 MeV, respectively. The improvement in the absorption coefficient (μ) values for the manufactured glass reduce the half-value thickness for the manufactured glass samples gets lowered as the μ values gets enhanced. Furthermore, the manufactured samples with high concentration of ZnO shows a superior ionized radiation shielding performance compared to other commercial borate-based glass. Hence, the manufactured sample coded BNZF-4 is a very wise choice for gamma shielding application in various sectors.

## Data Availability

The datasets used and/or analysed during the current study are available from the corresponding author on reasonable request.
